# Omics approaches for conservation biology research on the bivalve *Chamelea gallina*

**DOI:** 10.1038/s41598-020-75984-9

**Published:** 2020-11-05

**Authors:** Federica Carducci, Maria Assunta Biscotti, Emiliano Trucchi, Maria Elisa Giuliani, Stefania Gorbi, Alessandro Coluccelli, Marco Barucca, Adriana Canapa

**Affiliations:** Dipartimento di Scienze della Vita e dell’Ambiente, Università Politecnica delle Marche, Ancona, Italy

**Keywords:** Population genetics, Genetics, DNA sequencing, Next-generation sequencing, RNA sequencing, Molecular ecology

## Abstract

The striped venus (*Chamelea gallina*) is an important economic resource in the Mediterranean Basin; this species has exhibited a strong quantitative decline in the Adriatic Sea. The aim of this work was to provide a comprehensive view of the biological status of *C. gallina* to elucidate the bioecological characteristics and genetic diversity of wild populations. To the best of our knowledge, this investigation is the first to perform a multidisciplinary study on *C. gallina* based on two omics approaches integrated with histological, ecotoxicological, and chemical analyses and with the assessment of environmental parameters. The results obtained through RNA sequencing indicated that the striped venus has a notable ability to adapt to different environmental conditions. Moreover, the stock reduction exhibited by this species in the last 2 decades seems not to have negatively affected its genetic diversity. Indeed, the high level of genetic diversity that emerged from our ddRAD dataset analyses is ascribable to the high larval dispersal rate, which might have played a “compensatory role” on local fluctuations, conferring to this species a good adaptive potential to face the environmental perturbations. These findings may facilitate the efforts of conservation biologists to adopt ad hoc management plans for this fishery resource.

## Introduction

Commercially important marine species are frequently threatened by the effects of environmental changes, pollution, microbiological diseases, and overfishing, which may cause reductions in the size and genetic diversity of wild populations.

The development of omics sciences provides a remarkable contribution to species conservation biology, significantly increasing the ability of researchers to obtain insights into the molecular mechanisms adopted by species to cope environmental change^1^. The integration of data obtained from different omics approaches with ecotoxicological and environmental assessments has the advantage of creating a more holistic understanding of species, enabling researchers to develop specific tools to implement long-term management plans^2^.

*Chamelea gallina* (Linnaeus, 1758) is a mollusk species that is of considerable importance, both economically and ecologically. This bivalve, which belongs to the Veneridae family, is widely distributed along the eastern Atlantic coast, ranging from, Norway and the British Isles to the Iberian Peninsula, Morocco, the Madeira Islands and the Canary Islands^3^. This species is also observed in the Black Sea, Mediterranean waters^4^ and particular in the Adriatic Sea^5^. *C. gallina* is a gonochoristic species with a long spawning period, extending from April to August, in which egg emission takes place at intervals^6–12^. In the Adriatic Sea, the fishery of *C. gallina* represents one of the most important resources with approximately 15,000 t reported for the year 2018^13^, involving 670 fisheries with an annual turnover of approximately 100 million euros. For the Black Sea, the highest yield is reported in Turkey with approximately 40,000 t of annual catches^10^. *C. gallina*, similar to other marine bivalves, is also important in ecosystems, impacting nutrient cycling, creating and modifying habitats, and affecting food webs directly (i.e., prey) and indirectly (i.e., movement of nutrients and energy)^14^. Moreover, due to their filter-feeding abilities and their sessile mode of life, these bivalves play important roles as biosensors for pollution and other environmental changes in coastal waters, enabling the monitoring of the quality of the intertidal zones^15–18^. Several factors, including temperature variation, salinity, dissolved oxygen concentration in water and sediment^19–23^, presence of toxic substances of anthropogenic origin^24^, and overfishing^25^, might have induced a decrease in this fishery resource^12^.

In the Adriatic Sea, several mortality events due to sudden changes in the coastal environment (anoxia, river runoff, storm surges, pollution, and bacterial and/or viral infections) have strongly contributed to the decline of clam beds^12,26,27^ from approximately 35,000–15,000 t in the last 2 decades (FAO FishStat).

To date, the molecular resources available for *C. gallina* are the transcriptome published by Coppe et al.^28^ and gene expression analyses obtained by Milan and colleagues^26,27^, which detected differences between organisms sampled from two close sites with different local periodic mortality rates in the Italian Adriatic region of Abruzzo. Moreover, very little is known regarding the genetic substructure of *C. gallina*^29^.

Therefore, to ensure the long-term sustainability of fishery resources, further research elucidating the bioecological properties and the genetic diversity of wild *C. gallina* populations is required.

The utilization of high-throughput sequencing techniques, such as RNA sequencing (RNA-Seq) and double digest restriction-site associated DNA sequencing (ddRAD-Seq), is acquiring increasing recognition in the conservation biology research on sensitive and economically important species^30–32^. In particular, RNA-Seq is the favored sequencing technique to obtain knowledge on the functional response of organisms to environmental conditions. Indeed, in a multistress context, this technique enables us to depict the alteration of several genes and molecular pathways simultaneously in nonmodel organisms in response to stressful conditions^33–35^. ddRAD sequencing is one of the most powerful approaches to resolve fine-scale population structures compared with microsatellites, since ddRAD enables researchers to obtain thousands of markers^36,37^. This approach enables us to obtain a comprehensive view of genetic variability that represents a measure of the ability of species to adapt to environmental perturbations. Indeed, species showing low levels of genetic diversity are more prone to extinction. In line with these premises, this work attempted to provide, for the first time, a multidisciplinary strategy to elucidate the functional response of *C. gallina* to several biotic and abiotic factors and the genetic diversity level of the striped venus natural beds, as determined through RNA and ddRAD sequencing techniques. Moreover, to better contextualize the results obtained by combining omics approaches, we performed detailed histological, ecotoxicological, and chemical analyses and an assessment of environmental parameters.

## Results

### Transcriptomic analyses

**Figure 1:**
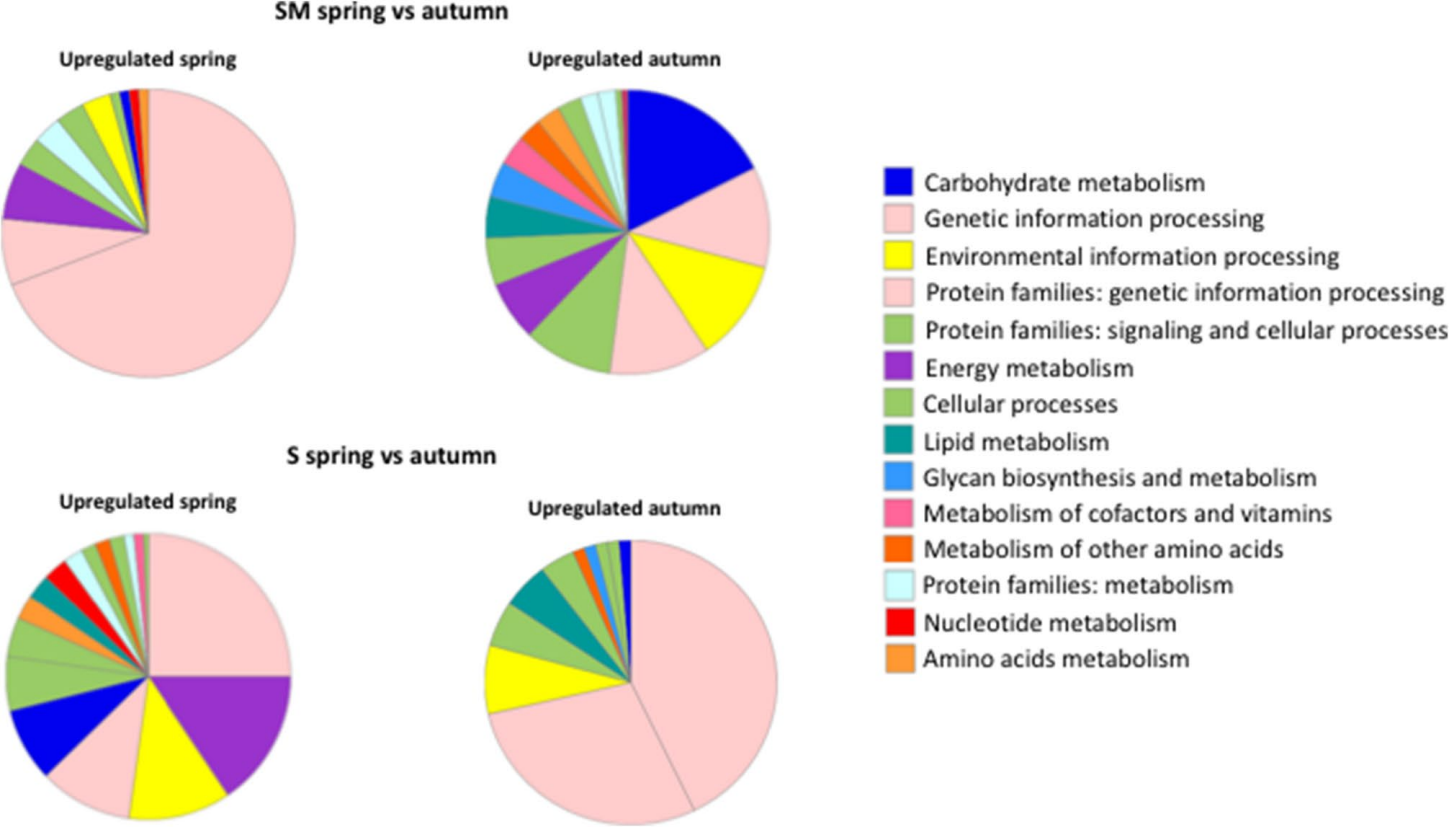
Graphical view of pathway enrichment analyses performed with KEGG BlastKOALA on transcriptomic data. Pathway enrichment analyses are shown^93–95^. In the upper side of the panel, comparisons between transcriptomic data obtained from clams collected at the two sampling times in SM. On the lower side of the panel, comparisons between transcriptomic data obtained from clams collected at the two sampling times in S.

RNA sequencing analyses were performed on individuals collected in spring and autumn 2018 from Senigallia (S) and Silvi Marina (SM), two sites to the north and south of Monte Conero, respectively, and belonging to two distinct biogeographic sectors. Differences in gene expression levels between the two sampling sites and sampling times were assessed by performing pairwise comparisons, as reported in Supplementary Fig. S1. The numbers of differentially expressed genes (DEGs) obtained using DESeq2 are shown in Supplementary Table S1. Principal component analyses (Supplementary Fig. S2) showed PC1 with a percentage of ≥ 69%, supporting a clear difference between the two sampling sites (Supplementary Fig. S2A and B) and between the two sampling periods (Supplementary Fig. S2C and D). In addition, the low percentage of PC2 suggested a good degree of concordance of the three biological replicates in each pairwise comparison (Supplementary Fig. S2). DEGs were investigated to identify the most significant biological processes and pathways involved in the functional response of *C. gallina* to continuously changing environmental conditions. Up- and downregulated genes were functionally annotated through pathway enrichment analyses for all pairwise comparisons performed in this study (Supplementary Fig. S1). For the S sampling site, between spring and autumn, genes upregulated in spring fell within *genetic information processing* (41 genes)*, energy metabolism* (29 genes)*, environmental information processing* (21 genes)*, protein families: genetic information processing* (21 genes)*, and carbohydrate metabolism* (15 genes). For this sampling site, genes upregulated in autumn were more closely related to *genetic information processing* and *protein families*: *genetic information processing* (53 genes) (Fig. 1). For the SM sampling site, these latter enriched pathways were more strongly represented within the upregulated genes in spring, while in autumn, *carbohydrate metabolism* (26 genes)*, genetic information processing* (18 genes)*, environmental information processing* (17 genes)*,* and *protein families: genetic information processing* (16 genes) were the most common. Furthermore, another comparison was performed between the two sampling sites in both sampling periods. Concerning the genes upregulated in spring for specimens sampled in S, *genetic information processing* (18 genes)*, energy metabolism* (12 genes)*, environmental information processing* (12 genes)*,* and *carbohydrate metabolism* (11 genes) were the most heavily represented, while for the upregulated genes in the SM sampling site, genes fell primarily within *genetic information processing* (21 genes) and *energy metabolism* (3 genes). An opposite pattern was observed when analyzing the enriched pathways related to the comparison between S and SM in autumn (Fig. 1).

### Focus on specific pathways

In analyzing data related to functional enrichment made using KEGG for the comparison between the two sampling sites and periods (Fig. 1), this research focused on genes involved in specific pathways.

#### Cellular processes

In KEGG pathway enrichment analysis, *cellular processes* included *transport and catabolism*, *cell growth and death*, *cellular community* (*eukaryotes* and *prokaryotes*), and *cell motility*.

**Figure 2:**
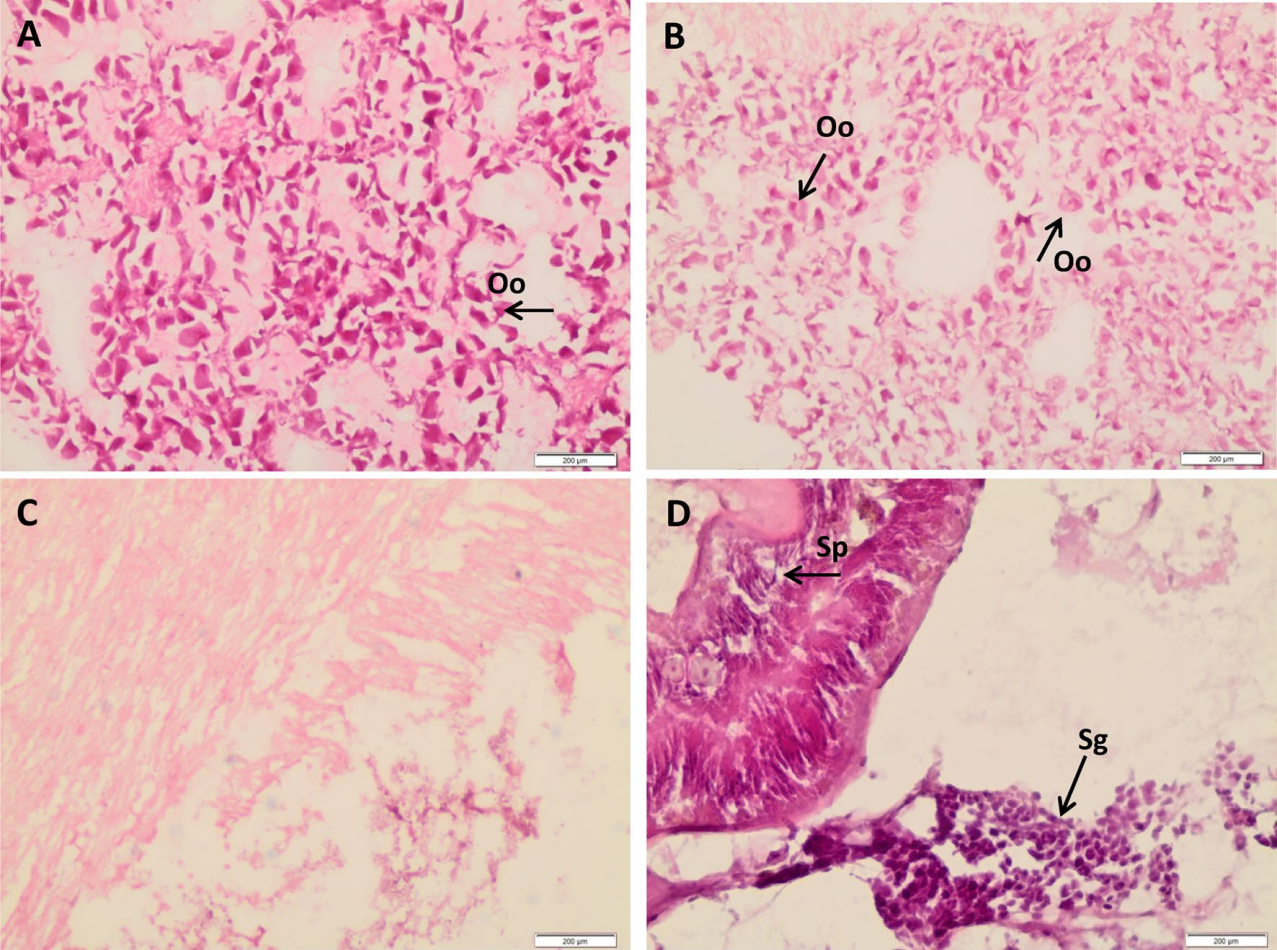
Histological analyses. *Chamelea gallina* gonadal sections made for the two sampling sites and periods: (**A**) S and (**B**) SM refer to the spring sampling period; (**C**) S and (**D**) SM refer to the autumn sampling period. Scale bars are 200 μm. Black arrows in (**A)** and (**B**) focus on oocytes (**Oo**) and their vitellogenic content. Black arrows in (**D**) focus on spermatozoa (**Sp**) and spermatogonia (**Sg**).

Functional annotation of the upregulated genes in the autumn period for the sampling site of SM enabled us to retrieve 22 entries that fall in the section of *cell growth and death*. In particular, the presence of the *SCF complex* (Skp-Cullin, F-box containing complex), *calmodulin*, *14-3-3ɛ protein*, *C-Myc binding protein, Dna J subfamily member 13*, and *spermatogenesis associated 4* among the upregulated gametogenesis-related genes was reported. At the same time, the absence of the *SCF complex* within the upregulated genes in functional enrichment analyses of the autumnal period at the S site was strongly in keeping with histological analyses showing gonadal inactivity (Fig. 2).

#### Energy, lipid, and carbohydrate metabolism

Energy metabolism plays a key role in the onset of reproduction^26,38^; thus, its analysis in bivalves, primary consumers of the aquatic food chain^39^, might help to elucidate their reproductive stage.

The variable level of food availability that depends on abiotic factors, such as solar radiation, salinity, and sea surface temperature^40^, influences the opportunistic behavior of *C. gallina*^38^.

**Figure 3:**
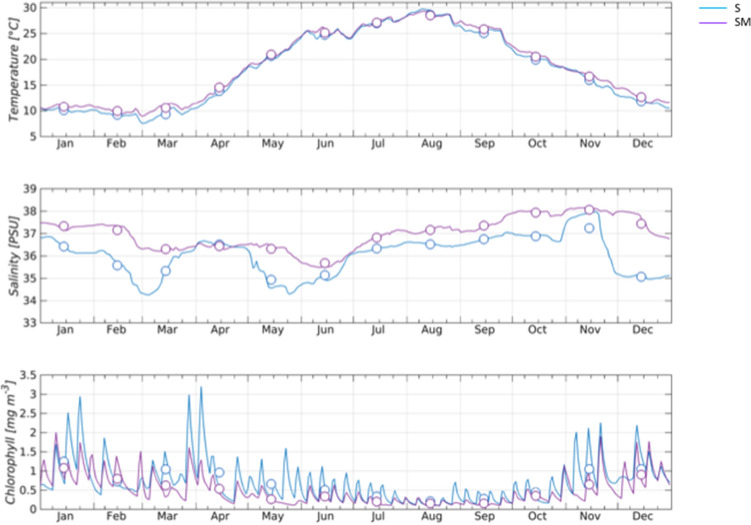
Time series of environmental parameters. Daily temperature (upper panel), salinity (middle) and chlorophyll (lower panel) at the sampling stations of S (blue line) and SM (purple line). Computed monthly means are superimposed as circles.

The results obtained from the DEG pathway enrichment analysis showed the presence within the upregulated genes of 33 genes related to *energy metabolism*, particularly of oxidative phosphorylation, together with the *carbohydrate metabolism* and *lipid metabolism* in the SM sampling site during the autumn period; these functional categories were less heavily represented in the upregulated genes of the spring period (Fig. 1). These pathways were closely connected with the employment of energetic resources in the production of ATP and *ready-to-use* energy for possible gamete production. Inverted and more pronounced, this trend of enriched pathways related to metabolism was observed in the comparison made for the DEGs of S specimens between the two sampling periods. In this case, the upregulated genes of the spring period were in *energy metabolism* (28 entries) and *carbohydrate metabolism* (15 entries), while in autumn, only 1 entry was in *carbohydrate metabolism*, and no entries were in *energy metabolism*. (Fig. 1). These findings may be explained by analyzing the data obtained in this study for the chlorophyll seasonal availability trend (Fig. 3), which suggests a higher food intake for S specimens collected in the spring period, which supports the activation of energy metabolism and earlier gametogenesis compared to SM specimens collected in the same period; this finding is in keeping with observations shown by the analysis of the *cellular processes* pathway.

### Ecotoxicological and chemical analyses

**Figure 4:**
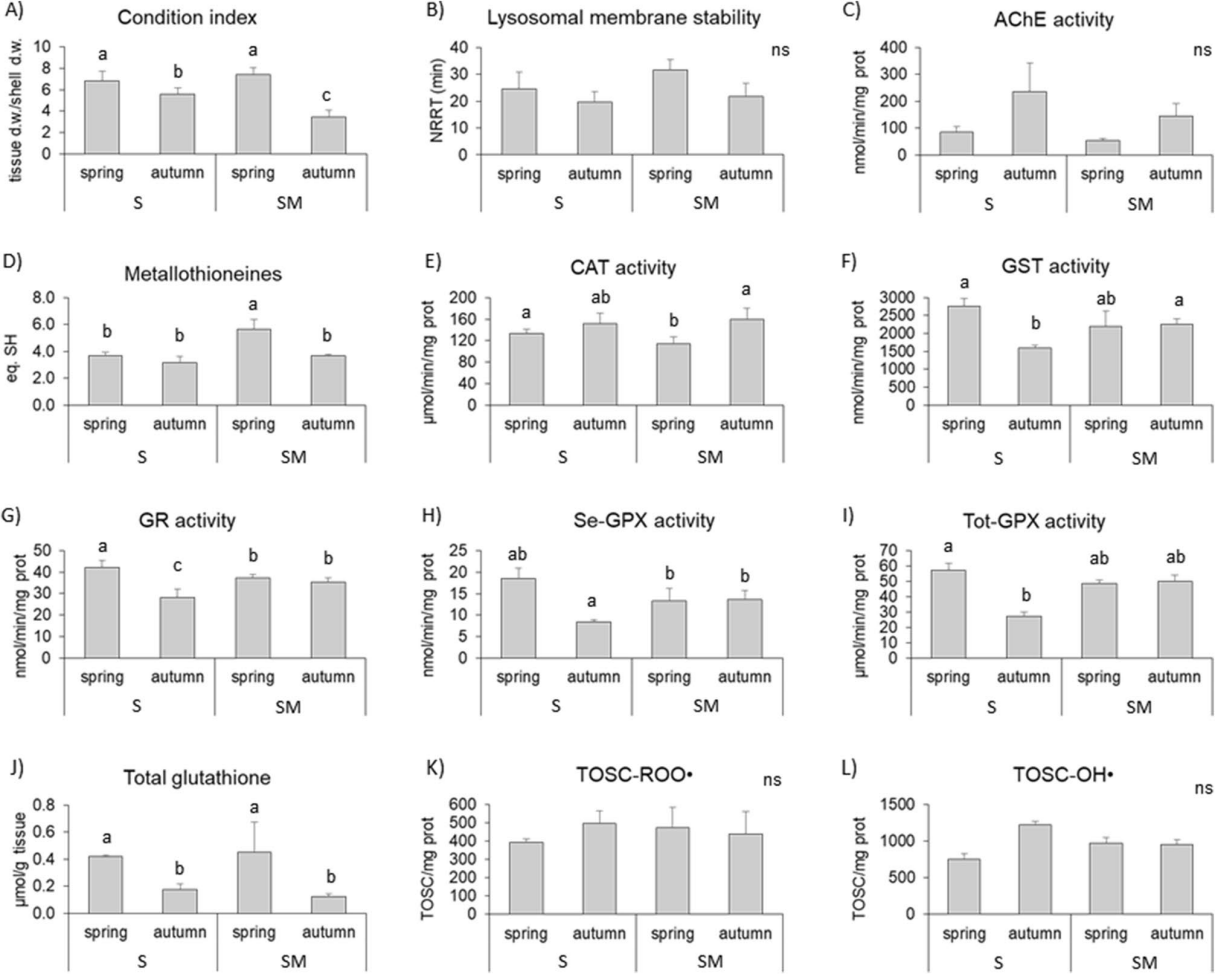
Biomarker responses analyzed in the clams *Chamelea gallina* collected in S and SM in spring and autumn 2018. (**A**) Condition Index of individuals (n = 30). (**B**) Lysosomal membrane stability in hemocytes (n = 5); NRRT = neutral red retention time. (**C**) Activity of acetylcholinesterase (AChE) in hemocytes (n = 5). (**D**–**I**) Activity of metallothioneines, catalase (CAT), glutathione S-transferases (GST), glutathione reductase (GR), Se-dependent glutathione peroxidases (Se-GPX) and total glutathione peroxidases (Tot-GPX) in the clam digestive gland (n = 5). (**J**) Total glutathione amount in digestive glands (n = 5). (**K**–**L**) Total oxyradical scavenging capacity (TOSC) against peroxyl radicals (ROO·) and hydroxyl radicals (OH·) measured in digestive glands (n = 5). All values are expressed as the mean ± standard deviation.

Clams collected in spring displayed a higher Condition Index (CI) than those sampled in autumn when a decrease was observed at SM compared to S (Fig. 4A). The lysosomal membrane stability in clam hemocytes exhibited no significant variations depending on season and site, although slightly lower values were detected in autumn than in spring (Fig. 4B).

The activity of acetylcholinesterase (AChE), as measured with a hemocytes, showed a nonsignificant increase in autumn compared to spring at both sites (Fig. 4C).

A significant difference in metallothionein content in the digestive glands was observed in clams collected at SM in spring compared to the values detected in the other sampling groups (Fig. 4D).

Antioxidants, measured in the digestive glands, showed fluctuations primarily related to the season. A slight increase was observed for CAT activity in autumn compared to spring, independent of the sampling site (Fig. 4E). The clams sampled in S exhibited significantly lower activities of all glutathione-dependent enzymes (GST, GR, Se-GPx, and total GPx) in autumn compared to those reported for the spring period; the values of the same enzymatic activities in clams from SM were constant during the two seasons, remaining at an intermediate level compared to those displayed in S clams (Fig. 4F–I). The amount of total glutathione was lower in autumn than in spring in clams from both sites (Fig. 4J). No significant differences were observed for the total oxyradical scavenging capacity (Fig. 4K–L).

The bioaccumulation analyses in clam tissues revealed limited variations in the different analyzed parameters (metals and PAHs), with the measured levels being similar to those typically observed in organisms from reference areas (Supplementary Table S2).

### Assessment of environmental parameters

Extracted timeseries span the year 2018. Data were provided as daily means for temperature, salinity, and chlorophyll (Fig. 3). Supplementary Tables S3 and S4 show the monthly and seasonal averages and the standard errors for the S and SM sampling sites, respectively. The results of the ANOVA and Tukey’s test show that there were no significant differences (*p* value > 0.05) between the two sampling stations in terms of seasonal temperature (Supplementary Table S5), whereas significant differences observed (*p* value < 0.05) for salinity in all seasons and for chlorophyll concentration in winter, spring and autumn. It can also be noted (Fig. 3) that salinity is generally lower at the S location due to the low salinity waters that are always present along the western coast, being related to river runoff^41^, particularly in the northern and middle part of the Adriatic Sea above the Monte Conero region. Analysis of the differences between the two stations on a monthly basis revealed that chlorophyll concentration is significantly different from March to May and in November when the S site was characterized by higher productivity (Fig. 3). The pairwise comparison among seasonal means of each parameter and at each sampling site (Supplementary Table S6) highlighted the expected differences in temperature and chlorophyll concentration, as these parameters have a clear seasonal pattern.

### Population genetics

**Figure 5:**
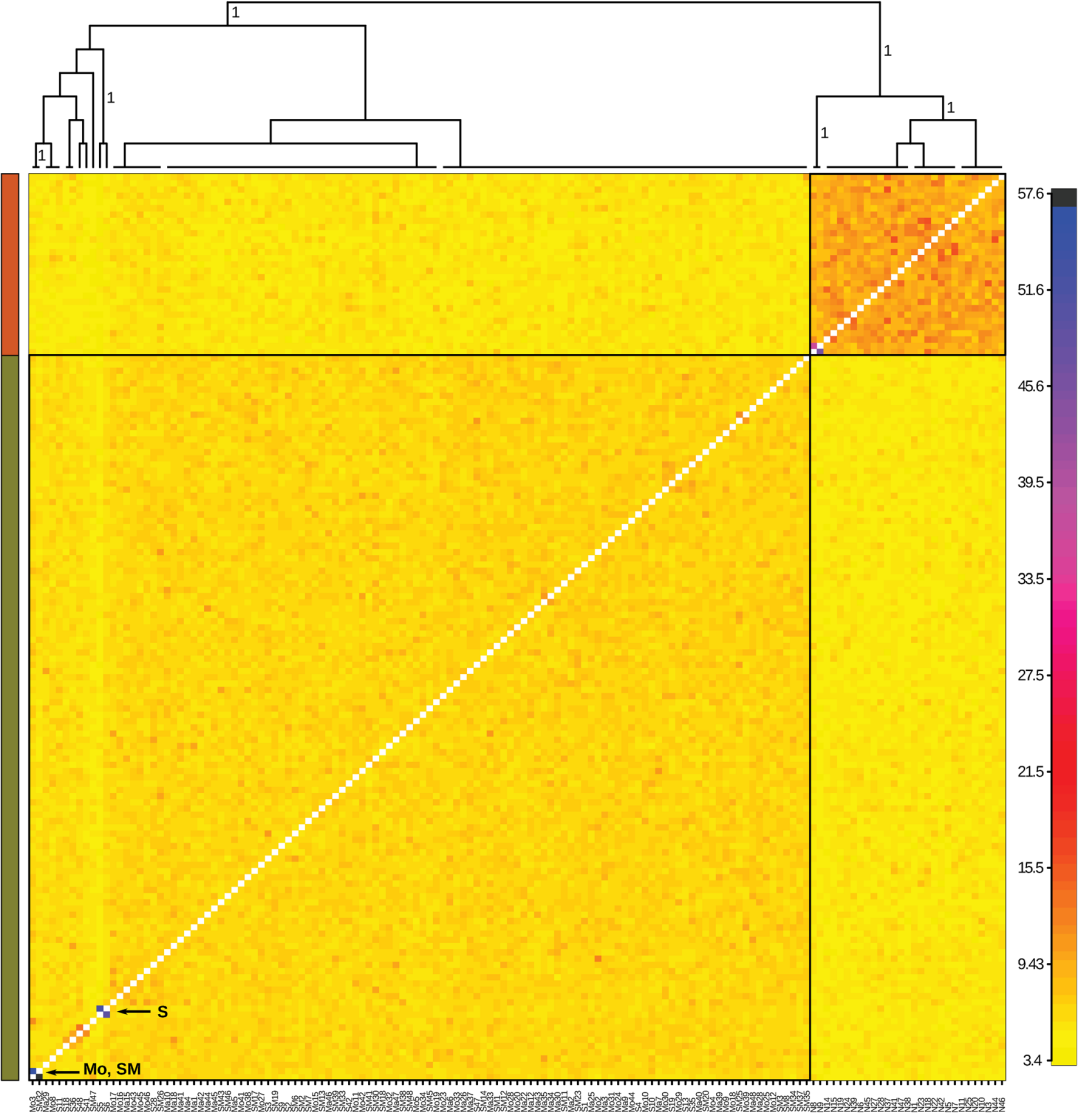
Structure of genomic diversity in striped venus from the Adriatic (green) and Tyrrhenian (orange) Seas. Coancestry matrix inferred by *fineRADstructure* using individuals with less than 25% missing data and loci with less than 45 SNPs (see also Figure S3b). The number of loci for which any two individuals are the closest relatives is coded according to the palette on the right. *FineRADstructure* Bayesian cladogram of the inferred (“natural” *k*) groups, with branch labels showing the posterior support for all of the included individuals to be assigned to that group, is shown at the top. The Adriatic population includes samples from Ma, Mo, S, and SM, whereas only N is the locality from the Tyrrhenian Sea. Few more-related-than-average individuals are highlighted.

**Figure 6:**
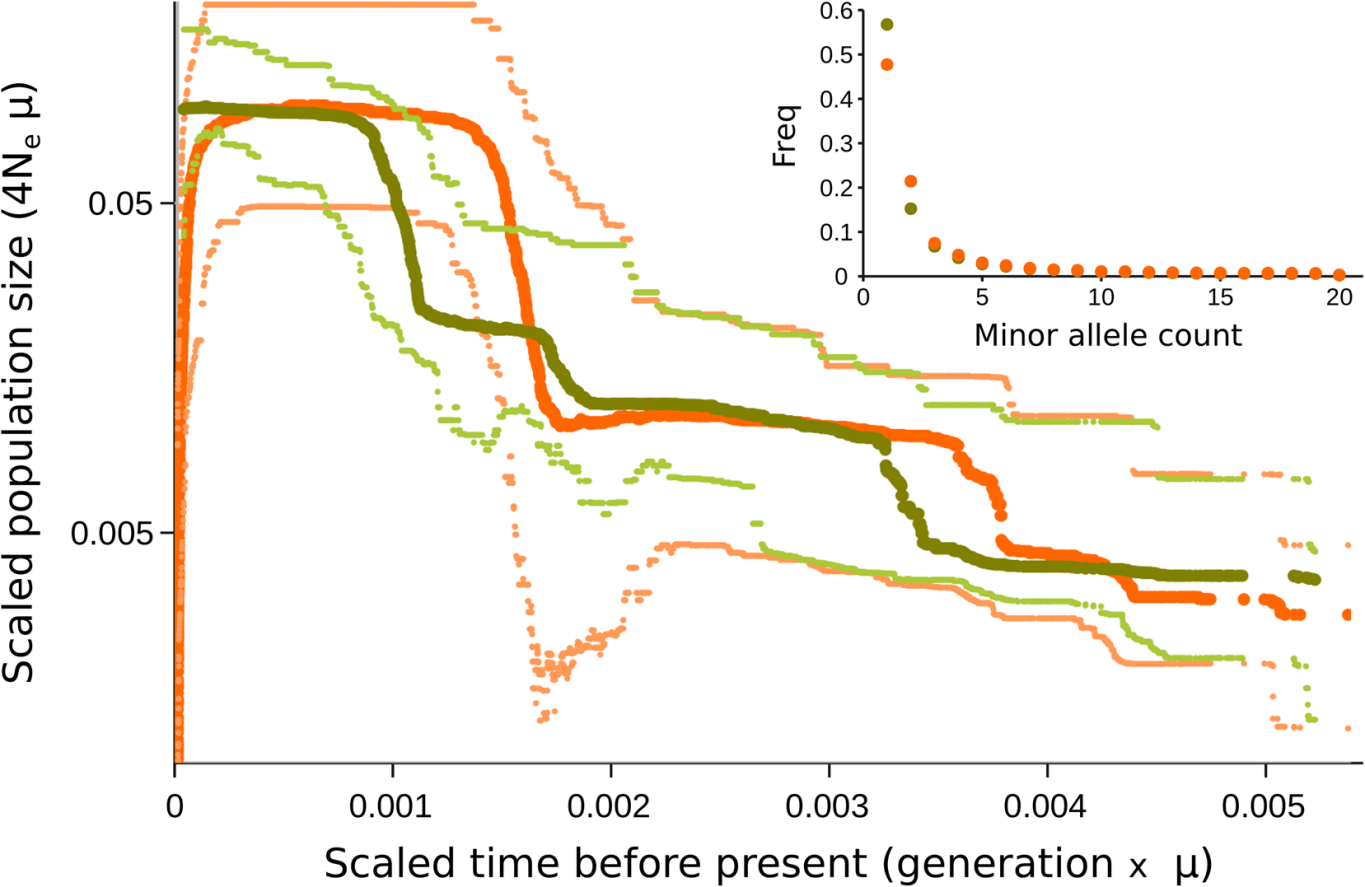
Demography of Adriatic (green) and Tyrrhenian (orange) populations, as inferred by a Stairway Plot. The minor allele site frequency spectrum for each population is shown in the top right (calculated on 20 individuals—see Methods). Mean (dark shade) and 95% confidence intervals (light shade). Effective population size (y-axis; log-scale) and time before present (x-axis) are scaled by the substitution rate.

A total of 10,543 paired-end loci with a mean length of ca. 230 bp and a total number of ca. 450,000 single nucleotide polymorphisms (SNPs) were de novo assembled and filtered by Stacks2.4 algorithms. Only ca. three hundred loci found a match in the GenBank nt database (1e−04), primarily with sequences identified as Bivalvia (Figure S3). After filtering out individuals with more than 50% missing loci and loci with more than 25% missing individuals, our dataset included 2004 loci and 155 individuals, showing a mean coverage per locus of ca. 60 X, a mean number of ca. 50 SNPs and 65 alleles per locus. The distribution of alleles and SNPs per locus appeared largely unimodal (a minor drop of approximately 45–50 SNPs per locus was further investigated; see Supplementary Fig. S3A and B). The marked difference in the amount of missing data between Tyrrhenian and Adriatic individuals is likely due to differential allele drop-out between the two groups, as supported by the principal component analysis (PCA)^42^ inferred on loci presence/absence (Supplementary Fig. S3C and D). Consistent with this finding, we inferred two populations with *fineRADstructure* corresponding to the Tyrrhenian and the Adriatic Sea (Fig. 5 and Supplementary Fig. S3E–H). In addition to a few individuals (mainly from S in the Adriatic Sea showing a higher coancestry with each other), the Adriatic population appeared to be fully panmictic, thereby suggesting a high level of gene flow among all sampled localities. The inferred genetic structure was robust to missing data (i.e., excluding individuals with more than 25% missing loci) and locus variability (i.e., including only loci with less than 45 SNPs). Most of the diversity within each population was attributable to low-frequency variants (Fig. 6); the average π per locus was 2.5e−4 and 2.3e−4 in Tyrrhenian and Adriatic populations, respectively, and the *F*_*ST*_ between the two populations was 0.075. The demographic reconstruction of the Adriatic population shows no sign of recent decline, as it is instead evident in the Tyrrhenian population (Fig. 6). Even if it is not possible to define a timewise framework for these reconstructions due to the lack of a reliable estimate of the substitution rate in this species, the comparison between the two histories is noteworthy.

## Discussion

The striped venus is an important economic resource in the Mediterranean Basin. However, it is known that in the Adriatic Sea, this species experienced a quantitative decline, and several factors have been suggested to be responsible for the reduction of natural clam beds.

In this study, for the first time, we employed two omics approaches based on high-throughput sequencing, the integration of which may be able to provide a comprehensive view of the biological status of the striped venus: RNA sequencing was performed to obtain information on the functional status of *C. gallina* in relation to several biotic and abiotic factors, and ddRAD sequencing was employed to assess if the genetic diversity level was enough to ensure the adaptive potential of the species to cope environmental perturbations to which it is continuously subjected. Moreover, ecotoxicological and chemical analyses, assessment of environmental parameters, and histological observations were performed to better contextualize the obtained data.

Transcriptomic analyses were performed on specimens collected in spring and autumn at two sampling sites located along the middle Adriatic coast of Italy. These two sites were chosen because of their economic importance in two maritime fishing districts. Moreover, the two sampling sites are located in the north and south of Monte Conero and belong to two different biogeographic sectors^43^. The promontory at this location influences water circulation patterns and the diffusion of runoff waters coming from the main northern Adriatic river (the Po River). The functional enrichment analyses on the DEGs obtained from the performed comparisons demonstrated a different representation of some pathways between specimens of the two sites and within the same site between specimens of the two sampling periods (Fig. 1).

Overall, most of the entries fall into the categories of *genetic information processing* and *protein families: genetic information processing*, whose genes are primarily related to the synthesis of ribosomal components. In particular, pathways referring to gametogenesis and reproduction, such as *cellular processes* and *energy, lipids,* and *carbohydrate metabolism*, are the most heavily represented in the spring sampling period of S and in the autumn period of SM.

Of significant interest was the presence of the *SCF complex* in the enriched pathway of specimens collected in the sampling site of SM in the autumnal period and absent within upregulated genes in functional enrichment analyses of the autumnal period in S. This gene is involved in the degradation of the Emi1 and Emi2 (early mitotic inhibitor 1 and 2) proteins, which are necessary for the progression of meiosis I and II, respectively^44^. Another upregulated gene identified in the same pathway was *calmodulin*, a mediator of cell cycle progression from meiosis I to meiosis II. In addition, *14*-*3*-*3ɛ protein* was found among the upregulated genes. This biomolecule belongs to a highly conserved protein family that is involved in a wide range of roles in cellular processes in all eukaryotes^45^, including the control of the cell cycle and the regulation of apoptosis^46^. Interestingly, the schematic representation of the *oocyte meiosis*-enriched pathway shows the *14*-*3*-*3ɛ isotype* in its dissociated form, essential for the progression of meiosis I. Indeed, the 14-3-3ɛ isotype binds the cell division cycle 25C (CDC25C) protein, thereby preventing cell cycle progression^47^.

Among the upregulated genes of sampling site S in the spring period and in SM in the autumnal period, male-specific genes involved in bivalve species gametogenesis^48^, such as *C-Myc binding protein*, *DnaJ subfamily member 13*, and *spermatogenesis associated 4*, were observed. All these findings suggest a timeline discrepancy in gonadal development in SM specimens compared to those collected at the S sampling site.

These observations were consistent with histological analyses, showing ongoing gametogenesis in SM specimens collected in the autumnal period in contrast to S clams. This finding is also in line with values observed for the Condition Index, a general indicator of the physiological status of organisms. In our case, lower values were recorded for specimens collected in the sampling site of SM in autumn. Lucas and Beninger (1985)^49^ have affirmed that a low value for this index may be the consequence of an intense biological effort that might be due to the gamete production process of the clams in this study.

Artigaud and colleagues in 2015^50^ reported an increase in the metabolic rate of animals, which was strictly related to glycolysis. In mollusks, an upregulation of the glycolytic pathway has been described during thermal acclimation in providing additional energy resources^51,52^. However, in our case study, the absence of substantial variations regarding surface water temperature enabled us to exclude the possibility that differences in metabolic rates observed between specimens of SM and S might be related to this type of environmental stressor. Therefore, variations in metabolic rates for *C. gallina* might be linked to the values observed for phytoplankton availability and, consequently, to food intake that could, in turn, have influenced the reproductive state in an opportunistic species.

In 2014, Joaquim and coworkers^38^ reported that in Portugal, gametogenesis in *C. gallina* occurs at the same time as the increase in food availability and is able to provide energy useful for this process^53,54^. Moreover, recent results^11,38^ challenge the canonical evidence reported for the reproductive cycle of *C. gallina,* in which two peaks of gamete emission in April and October were described^6–9^. Indeed, analyses performed on natural populations of *C*. *gallina* living in the Gulf of Cadíz, as reported by Delgado and colleagues^11^, demonstrated a long reproductive period between March and September with a recovery of gametogenesis in November related to the high peak of chlorophyll-a availability observed in October. In addition, an intragonadal and interindividual asynchrony has been reported for the striped venus. These observations are in keeping with the timeline discrepancy in the reproduction of *C. gallina* specimens between the S and SM sites. This timeline incongruence might be consistent with two possible scenarios: gametogenesis in SM clams is delayed and thus covers a shifted forward period compared to S specimens; either SM clams experienced an additional peak of gamete emission in the autumnal period. Ecotoxicological and chemical analyses supported our interpretation of transcriptomic data. Although it is known that cellular responses can be affected by environmental contaminants, the determination of chemical parameters in the clam tissues enabled us to exclude pollutants as possibly causes of observed variations in both transcriptomics and in biomarker responses. Indeed, detected bioaccumulation values were below regulatory limits for bivalve mollusks (where present) and similar to those found in *C. gallina* inhabiting nonpolluted areas (EC 1881/2006; EC 835/2011)^24,55^.

It is known for several bivalve species that biological parameters undergo seasonal variations in natural populations not exposed to contaminated conditions; hence, the differences shown from biomarker analyses in marine organisms employed as bioindicators of environmental quality might be related to a different physiological state^56–58^. The results obtained in the present study highlighted that in *C. gallina,* the biological responses utilized as biomarkers were modulated by biotic and abiotic seasonal factors, including salinity, temperature, food availability and reproductive status, confirming again the influence of these factors on biomarker modulation in sentinel marine species^56,59,60^. In particular, in S clams, the higher levels of glutathione and of the activities of all glutathione-dependent antioxidant enzymes (GST, GR, Se-GPx, and total GPx) in the spring period can be related to an increased metabolic rate, as also indicated by the upregulation of *energy metabolism* genes, the higher phytoplankton availability and the active gonadic status. In contrast, only small variations in biological responses (in particular, CAT activity and levels of GSH and MT) were observed in SM clams between spring and autumn seasons, although a certain difference was observed at the transcriptomic level (upregulation of *energy metabolism* genes in autumn). This finding could be interpreted as an indication that transcriptional responses occur earlier, and they are translated into a functional effect later.

The findings discussed to this point indicate an intriguing ability of the striped venus to adapt to different environmental conditions. It is known that the adaptive potential of species to cope environmental perturbations is positively correlated with genetic diversity. Therefore, the assessment of the genetic diversity level of the striped venus natural beds has been performed to confirm that the quantitative decline experienced by *C. gallina* in the last 2 decades did not negatively affect the genetic variability and, consequently, its ability to respond to modified environments.

The results showed that striped venus individuals sampled at five different localities in the Adriatic Sea, spanning over 600 km (from Monfalcone (Mo) to SM), are all part of a largely panmictic population (Fig. 5), as also reported in species with high larval dispersal rates^61–63^ and in species with higher adult dispersal rates, such as squid and cuttlefish^64^. Moreover, the *F*_*ST*_ between Adriatic (altogether) and Tyrrhenian localities is high compared to the differentiation recorded in the Mediterranean Basin for other marine mollusks, such as the wedge clam^65^, the octopus^66,67^ and the bivalve *Pecten jacobaeus*^37^, or more mobile fish species^68–70^.

The high genetic diversity recorded together with the existence of a unique population in the Adriatic Sea confers to this species a higher responsiveness to local environmental perturbations (e.g., overfishing, pollution, and mortality due to microbiological infections), as reported for invertebrate marine species^71–73^. Genetic interconnection due to larval dispersion between Adriatic sites represents a fundamental counterforce against local population size decline, thereby increasing the ability of population persistence^74^. Larval dispersion seems to have played a less important role due to the species’ limited and patchy distribution along the Italian Tyrrhenian coast and to the separation from the Adriatic population. Accurate comparisons on diversity and structure are, however, limited because of the scarcity of genomic studies in Mediterranean marine invertebrates^75^.

Even though the striped venus fishery recorded a marked decline over the last 20 years^12,76^, our demographic reconstruction showed no signature of recent decline in the Adriatic population size (Fig. 6). However, the time and population size scale of our demographic inference should be considered with caution, as we do not have precise information on the mutation rate in this organism. In fact, the extremely high genetic diversity in our ddRAD-Seq dataset could be explained by a very high effective population size and/or by a very rapid mutation rate^75^. At any rate, the contrast between the recent demography of the striped venus population from the Adriatic and the population from Naples, where a decline in population size has also been directly observed^77^, is evident (Fig. 6).

Moreover, genetic structure in sessile marine organisms is expected to be related to the length and dispersal capacity of the larval stage^63^ and, not negligibly, to the oceanographic barriers determined by water circulations^62,78–80^. In particular, in the south of the Adriatic Sea (the Strait of Otranto), a circular current of the upper water masses represents a possible constraint of *C. gallina* pelagic larval migration, enabling the isolation of the Adriatic one from the other Mediterranean populations. This situation has also been observed in other marine species^63,69^. The cause of the differentiation of the Adriatic *C. gallina* population from that of the Tyrrhenian Sea might also be attributed to the geodynamic evolution of the Mediterranean Basin. The oldest fossil records of the venus clam date to the Oligocene^80^ and the Messinian Stage in the late Miocene. Most likely, the deep biota underwent extinction, while the shallow-water biota may have survived this drastic event^81–84^.

## Conclusions

This study provides a multidisciplinary approach that is able to provide an exhaustive view of the biological status of striped venus. Comparisons of transcriptomic and histological data between *C. gallina* specimens collected in two different sites of the Italian middle Adriatic coast showed a substantial temporal difference in gonadal development and gamete emission. This characteristic is likely due to the “opportunistic” behavior of *C. gallina*, which is able to exploit local fluctuations in nutrient availability. Ecotoxicological and chemical investigations excluded the effects of pollution and anthropic factors, suggesting a good healthy status of the striped venus. The high genetic diversity level that emerged from our ddRAD dataset analyses suggests that stock reduction has not negatively affected *C. gallina* natural beds. Indeed, the high larval dispersal rate might have played a “compensatory role” on local fluctuations, conferring a good adaptive potential of this species to face the environmental perturbations to which it is continuously subjected.

Therefore, elucidating the bioecological properties and the genetic diversity of wild populations is a *conditio sine qua non* in the field of conservation biology to adopt ad hoc management plans of fishery resources, which may also result in positive effects on the fishing economy.

## Materials and methods

This study was conducted on invertebrates (molluscs), not subjected to specific permissions since the sampling area is not privately owned or protected and did not involve endangered or protected species.

### Experimental sampling plan

Clams *Chamelea gallina* of similar size and weight (25.4 ± 2.1 mm shell length; 755 ± 92 mg body wet weight) were collected by hydraulic dredge, in different sites and periods.

**Figure 7:**
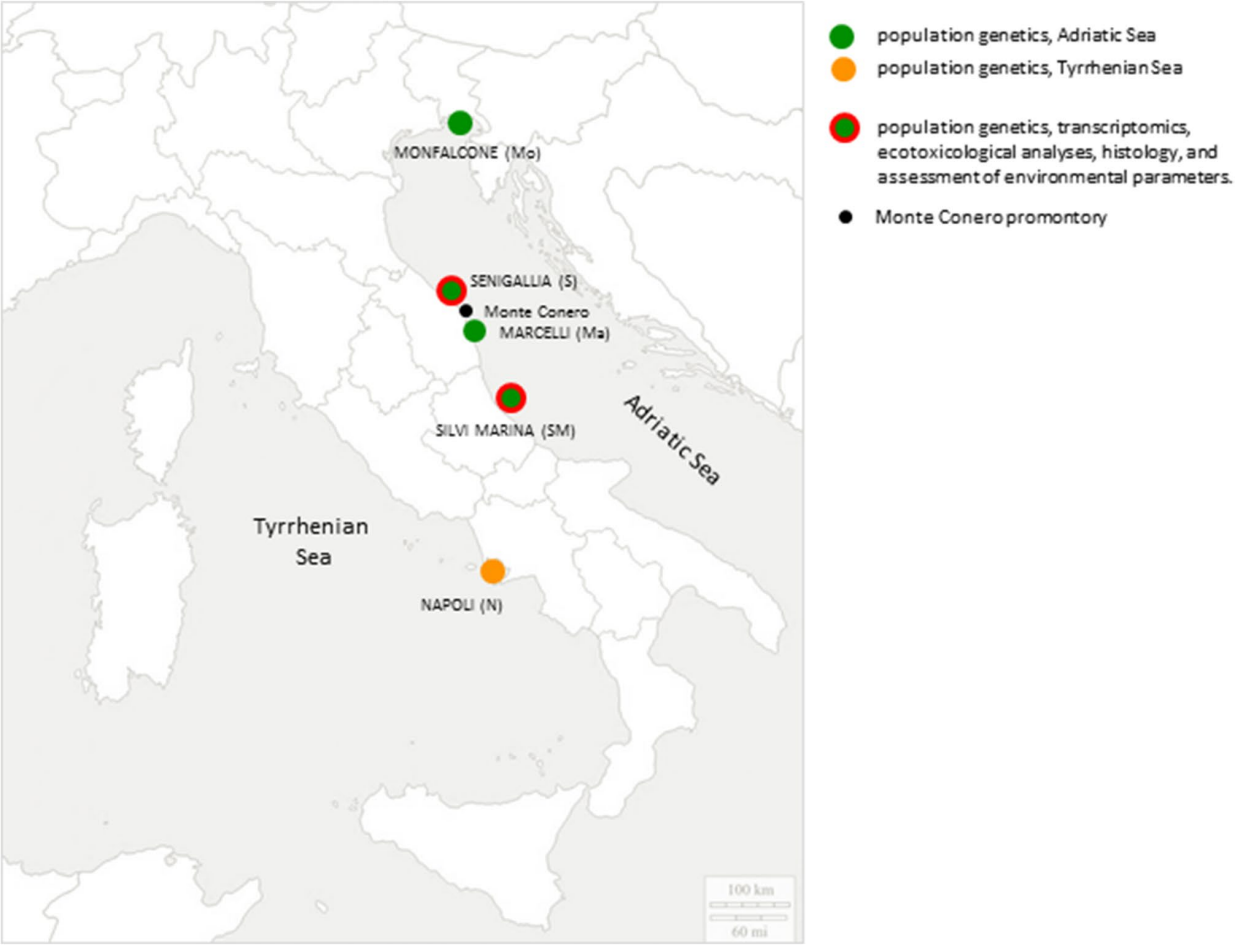
Sampling and experimental plan. Specimens collected from Monfalcone (Mo), Senigallia (S), Marcelli (Ma), Silvi Marina (SM), and Napoli (N) sampling sites were investigated through population genetics (dark green dots: sampling sites located in the Adriatic Sea; orange dot: sampling site located in the Tyrrhenian Sea; black dot: Monte Conero promontory). Transcriptomics, histology, ecotoxicological analyses, and assessment of environmental parameters were performed on samples collected from S and SM sampling sites (red circled dark green dots).

For population genetics analyses, 155 specimens were sampled from five sites (Fig. 7, coordinates Marcelli of Numana (Ma): 43° 31′ 03.60″ N/013° 39′ 16.68″ E, Monfalcone (Mo): 45° 42′ 36.00″ N/013° 12′ 30.60″ E, Naples (N): 40° 49′ 12.00″ N/014° 02′ 24.00″ E, Senigallia (S): 43° 41′ 01.38″ N/013° 17′ 48.72″ E, Silvi Marina (SM): 42° 33′ 19.92″ N/014° 08′ 01.26″ E).

Concerning transcriptomic, ecotoxicological, chemical, and histological analyses, specimens of the two sampling sites, S and SM, were collected in late spring and autumn 2018. In particular, for transcriptomic analyses, three pools of digestive glands dissected from 10 specimens were analysed for each sampling site and period; for histological analyses 20 specimens for each sampling site were examined to determine the stage of gonadal development. Ecotoxicological and chemical analyses required a total of 235 specimens for each sampling group: whole tissue was collected from 30 individuals for the calculation of Condition Index (CI = [dry tissue weight (g)/dry shell weight (g)] × 100)^85^; fresh hemolymph was taken from 5 specimens for the analysis of lysosomal membrane stability; 5 pools of digestive gland and hemolymph (10 organisms per pool) were frozen in liquid nitrogen and stored at − 80 °C for ecotoxicological analyses (biomarkers); 3 pools of whole tissues (50 organisms per pool) were collected for chemical analyses of bioaccumulation.

### Transcriptomic analyses: sequencing and bioinformatics analyses

Total RNA was extracted using Trizol reagent (Invitrogen, ThermoFisher Scientific) and purity and quantity were checked using Qubit 4.0 fluorometer (ThermoFisher Scientific). RNA integrity was evaluated by Agilent 2100 Bioanalyzer (Agilent technologies, Santa Clara, CA). RNAs were sent to IGA Technology Service (Udine, Italy; https://igatechnology.com) where TruSeq Stranded mRNA Sample Prep kit (Illumina, San Diego, CA) was used for library preparation following the manufacturer’s instructions, starting with 1 µg of good quality RNA (R.I.N > 7) as input. The poly-A mRNA was fragmented 3 min at 94 °C and every purification step was performed by using 1 × Agencourt AMPure XP beads. Libraries were then processed with Illumina cBot for cluster generation on the flowcell, following the manufacturer’s instructions and sequenced on 2 × 125 bp paired-end mode on HiSeq2500 (Illumina, San Diego, CA).

### Gene expression analysis

The Bcl2Fastq 2.0.2 version of the Illumina pipeline was used to process raw data for both format conversion and de-multiplexing. Removing lower quality bases and adapters was carried out using ERNE^86^ and Cutadapt^87^ softwares. Trimmed RNA-Seq reads of each sample were mapped on the *C. gallina* reference transcriptome^28^ using STAR^88^. After performing the assembly and quantification of the full-length transcripts using StringTie^89^, the count of overlapping reads was performed with HTseq count^90^. DESeq2^91^ was used to perform comparisons between expression levels of genes and transcripts as follow: S spring versus S autumn; SM spring versus SM autumn; S spring versus SM spring; S autumn versus SM autumn (Supplementary Fig. S1).

Contigs obtained from comparative analyses conducted using DESeq2 were used to perform functional annotation and pathway enrichment analyses. From each comparison, genes were filtered using the Benjamini–Hochberg adjusted *p *value < 0.01, genes showing a negative log_2_ fold change value were considered as downregulated while those with a positive log_2_ fold change value were considered as upregulated. CLC genomic workbench v12.0 (Qiagen, Hilden, Germany) was then used to perform domain search in PFAM database v32^92^. Functional enrichment analysis was performed with KEGG BlastKOALA^93–95^ (https://www.kegg.jp/blastkoala/).

### Hystological analyses

Gonadal tissues of specimens from S and SM were histologically examined to determine the stage of gonadal development. After embedding in Killik (Bio-Optica, Milano, Italy) medium, sections of 8–10 μm were obtained using Leica cryostat (Wetzlar, Germany). The histology prepared slides were stained with haematoxylin and eosin method and inspected using the optical light microscope (Olympus Co., Tokyo, Japan).

### Ecotoxicological and chemical analyses

Ecotoxicological analyses (biomarker) analyses were carried out through standardized procedures and included: Condition Index (CI) calculation; analysis of lysosomal membrane stability in the hemocytes; spectrophotometric determination of acetylcholinesterase activity in the hemolymph; spectrophotometric determination of single antioxidants (catalase, glutathione S-transferases, glutathione peroxidases and glutathione reductase activity; total glutathione content) and gas-chromatographic assay of total antioxidant scavenging capacity toward peroxyl and hydroxyl radicals^96,97^; metallothioneins level^98^.

Chemical analyses of trace metals (As, Cd, Cr, Cu, Fe, Hg, Mn, Ni, Pb, V, Zn) and polycyclic aromatic hydrocarbons (PAHs), both low molecular weight (naphthalene, acenaphthylene, 1-metylnaphthalene, 2-metylnaphthalene, acenaphthene, fluorene, phenanthrene, anthracene) and high molecular weight (fluoranthene, pyrene, benzo[a]anthracene, chrysene, 7,12-dimetylbenzo[a]anthracene, benzo[b]fluoranthene, benzo[k]fluoranthene, benzo[a]pyrene, dibenzo[a,h]anthracene, benzo[g,h,i]perylene, indeno[1,2,3,c,d]pyrene) were performed on whole clam tissues by conventional procedures base on gas-chromatography with flame ionization detector, HPLC with fluorimetric detection, and atomic absorption spectrophotometry^99^.

Details of analytical procedures for both ecotoxicological and chemical analyses are given in Supplementary File S1.

Statistical comparison of biological parameters was performed through the analysis of variance (ANOVA) with a level of significance set at *p* < 0.05, and the post hoc comparison Newman–Keuls. The analyses were performed with the R software.

### Assessment of environmental parameters

Historical timeseries of temperature, salinity and chlorophyll concentration are taken from the output of hydrodynamics and biogeochemical models of the Mediterranean Sea, publicly available on E.U. Copernicus Marine Service Information (https://marine.copernicus.eu). Temperature and salinity were extracted from the Mediterranean Sea Physics Analysis and Forecast dataset^100^ which nowadays represents the best available estimate of the physical environment and dynamics of the whole Mediterranean Sea. Chlorophyll timeseries were extracted from the Mediterranean Sea Biogeochemistry Analysis and Forecast dataset^101^ which is produced by a biogeochemical fluxes model, named MedBFM, driven by physical forcing fields from the aforementioned analysis and forecast dataset. Extracted timeseries span the year 2018. Data were provided as daily means for temperature, salinity, and chlorophyll. The model grid sea-points nearest to S and SM sampling stations were chosen and data were extracted at the sea-floor level. Seasonal means of the year 2018 were calculated and further processed with the aim to check if the two sampling stations exhibit significant differences in terms of monthly and seasonal means of the parameters taken into account. The assessment was performed by applying a one-way analysis of variance (ANOVA) and a Tukey’s test as post-hoc pairwise comparison. The null hypothesis stated that there were no differences in terms of temperature, salinity or chlorophyll concentration between the two sampling stations. The level of significance was set at 0.05. The software Unistat 10 was used to perform the analysis.

### Population genetics: sequencing and bioinformatics analyses

Total DNA extraction from foot tissue was performed for 48 individuals of each sampling site (Fig. 7) using DNeasy Blood and Tissue Kit (Qiagen). Information on the sex of animals were not recorded. After total extraction, purity and quantity of total DNA extracted were checked using Qubit 4.0 fluorometer (Thermo Fisher Scientific). DNA samples were sent to IGA Technology Service (Udine, Italy; https://igatechnology.com) where ddRAD libraries were produced using IGATech custom protocol, with minor modifications of Peterson’s and colleagues (2012)^102^. Libraries were constructed using *Sph*I and *Hin*dIII restriction enzymes and sequenced with V4 chemistry paired end 125 bp mode on HiSeq2500 instrument (Illumina, San Diego, CA).

Raw reads were demultiplexed by *process_radtags* in Stacks 2.4^103^ with default settings. Paired-end reads from all individuals were de novo assembled using the script *denovo_map.pl* in the Stacks package, allowing for maximum two mismatches between stacks to call a locus within and among individuals (-M 2 and -n 2), disabling haplotype calling from secondary reads (–H) and setting the maximum number of stacks at locus to 2 (–max_locus_stacks). The script *populations* in the Stacks package was employed to discard loci found in less than 40% of individuals across all populations (− R = 0.4) and with observed heterozygosity higher than 0.8 (–max-obs-het). Before any downstream analysis, our dataset for contamination by non-endogenous DNA was tested. Blast^104^ was used to map the consensus sequence of each locus to *nt* database (downloaded on 14/09/19) applying an *epsilon* threshold of 1e−04. Blast results were taxonomically summarized using MEGAN^105^ (Supplementary Fig. S4). The catalog of loci was then further reduced, first excluding individuals with more the 50% missing loci and then loci with more than 25% missing individuals.

The scripts *Stacks2fineRAD.py* included in the *fineRADstructure* package^106^ was used to estimate the distribution of alleles and SNPs per locus and of the missing data per individual. As ddRAD is prone to batch effect due to small deviations among libraries at the size selection step, we checked whether missing data were driving any library-based structure. A PCA analysis converting all loci in presence/absence data was performed using the same script as before. Beside any batch effect, allele drop-out due to genetic differentiation among individuals was also evident with this analysis. To investigate the genetic structure in our sample and to test for its robustness to missing data and number of SNPs per locus, *fineRADstructure* after filtering the data with different settings in *Stacks2fineRAD.py* was used. In particular, the analysis with a full dataset (2004 loci and 155 individuals) was run, selecting only loci with less than 45 SNPs (918 loci), selecting individuals with less than 25% missing data (147 individuals), and the combination of both filters (918 loci and 145 individuals–individuals were filtered after the loci).

The folded site frequency spectrum (SFS) was calculated in each of the two populations inferred by *fineRADstructure* using a custom python script (Supplementary File S2), normalized over 20 individuals to take into account the sampling difference between the two populations, and not allowing for missing data. Average number of nucleotide pairwise differences (π) per locus was calculated within each population using *vcftools*^107^ and considering an average locus length of 230 bp. Weighted *F*_*ST*_ between the two populations was also calculated using *vcftools*.

To separately infer the past demographic histories of the two populations, we used the SFS as calculated before in Stairway Plot^108^, a model-free method to infer the variation in past coalescent rate within a population through the maximization of the composite likelihood of the site frequency spectrum. The total number of observed sites was calculated as the number of loci times an average locus length of 230 bp and all bins of the SFS were used given the high confidence in heterozygous sites calling due to the high coverage per locus. As mutation rate is not known in this species, time and population size of the demographic reconstruction are given as scaled by the mutation rate.

## Supplementary information

Supplementary Information 1.

Supplementary Information 2.

Supplementary Information 3.

Supplementary Information 4.

Supplementary Information 5.

Supplementary Information 6.

Supplementary Information 7.

Supplementary Information 8.

Supplementary Information 9.

Supplementary Information 10.

Supplementary Information 11.

Supplementary Information 12.

Supplementary Information 13.

## Data Availability

ddRAD and RNA-Seq dara are deposited in SRA database (BioProject PRJNA625642 and PRJNA625643, respectively). Historical timeseries of temperature, salinity and chlorophyll concentration are publicly available from the output of hydrodynamics and biogeochemical models of the Mediterranean Sea, on E.U. Copernicus Marine Service Information (https://marine.copernicus.eu).
