# Supplementary figures and images for: Omics approaches for conservation biology research on the bivalve Chamelea gallina
Source: Sci Rep. 2020 Nov 5;10:19177. doi: 10.1038/s41598-020-75984-9 (PMC7645701; doi:10.1038/s41598-020-75984-9)

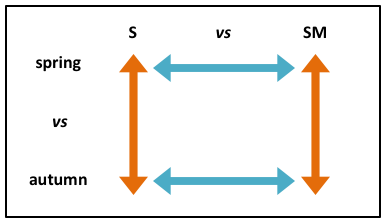

Supplement: Supplementary file 1 — Supplementary Information 1. [file 41598_2020_75984_MOESM1_ESM.tiff]

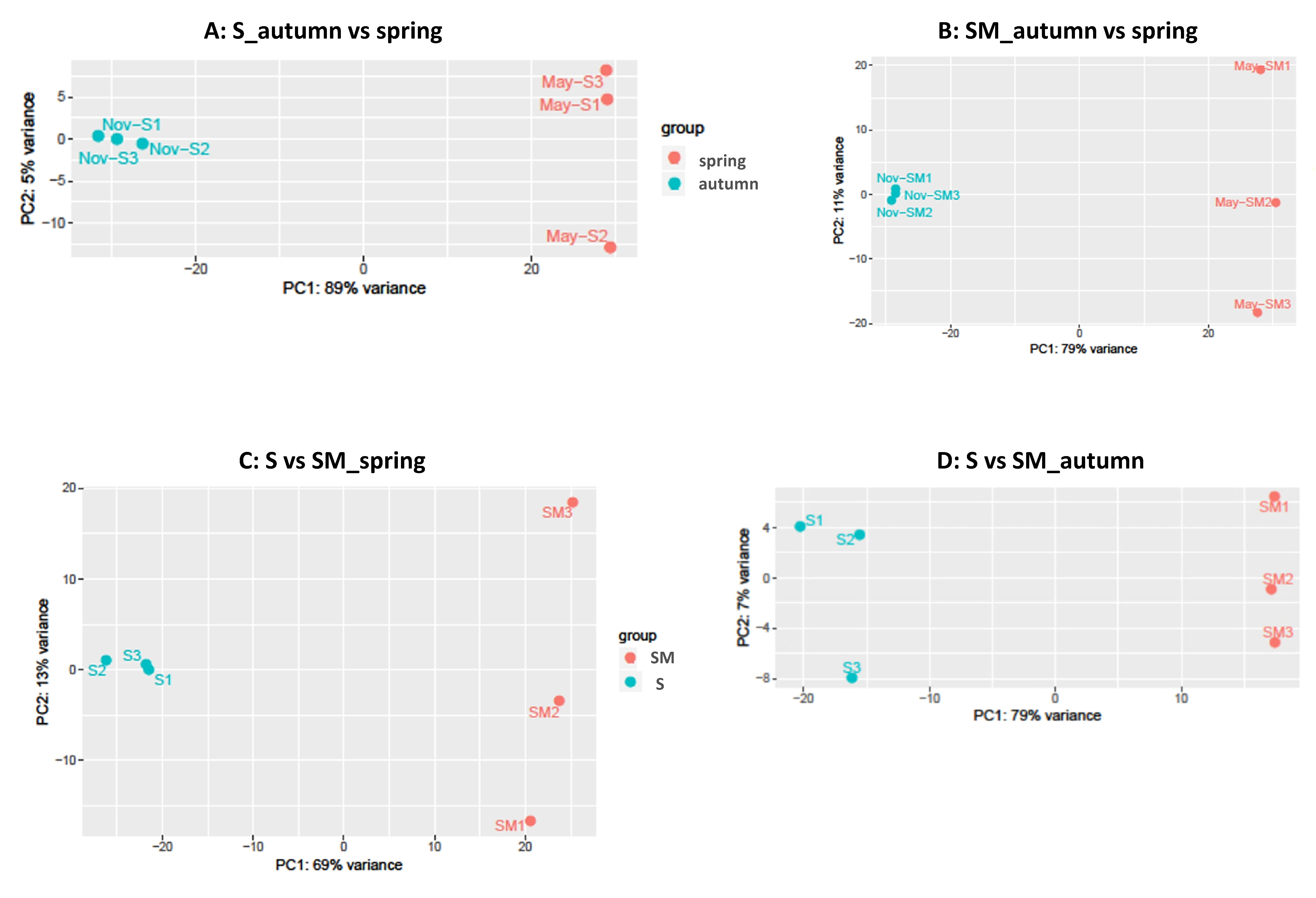

Supplement: Supplementary file 2 — Supplementary Information 2. [file 41598_2020_75984_MOESM2_ESM.tif]

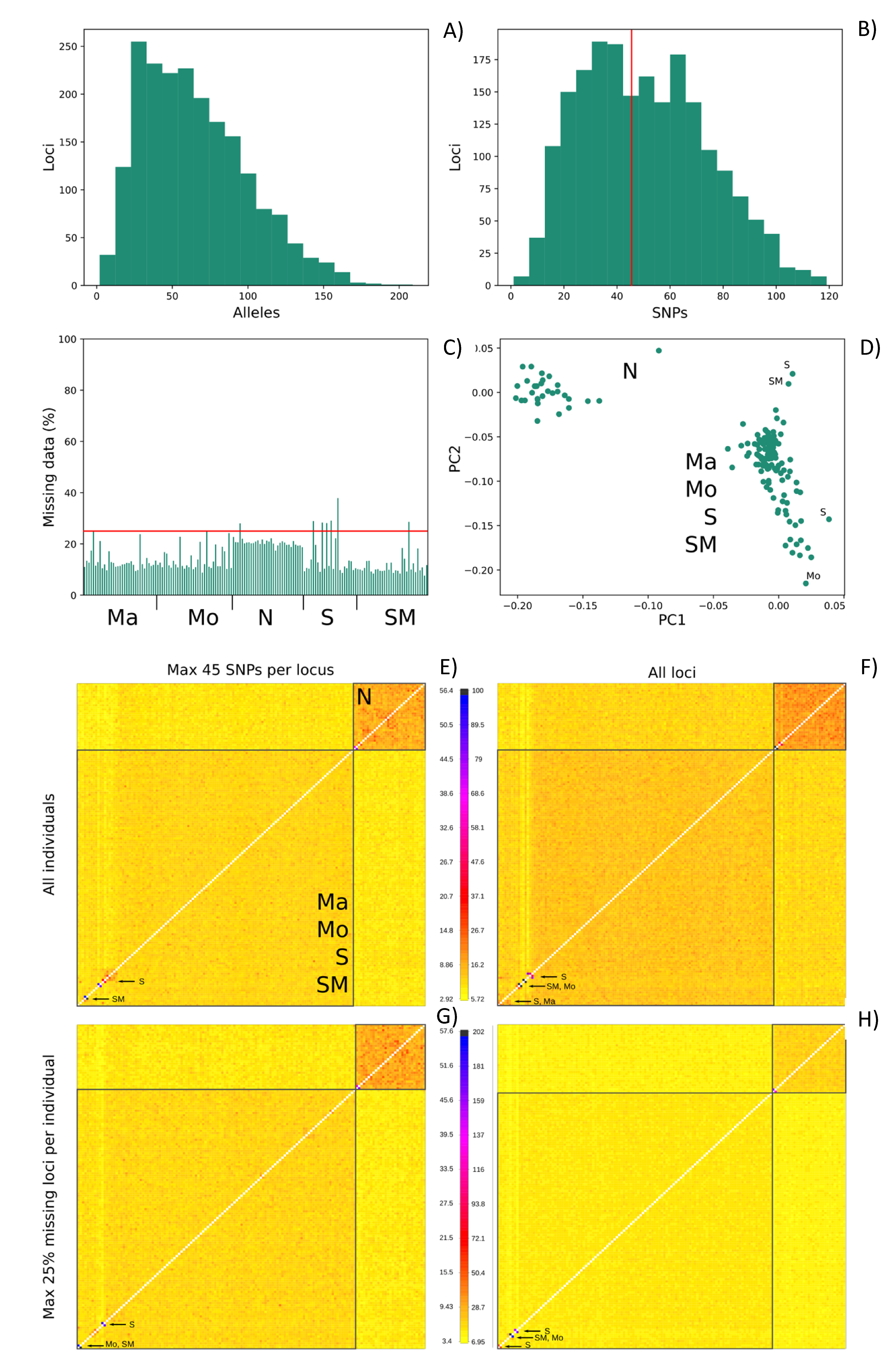

Supplement: Supplementary file 3 — Supplementary Information 3. [file 41598_2020_75984_MOESM3_ESM.tif]

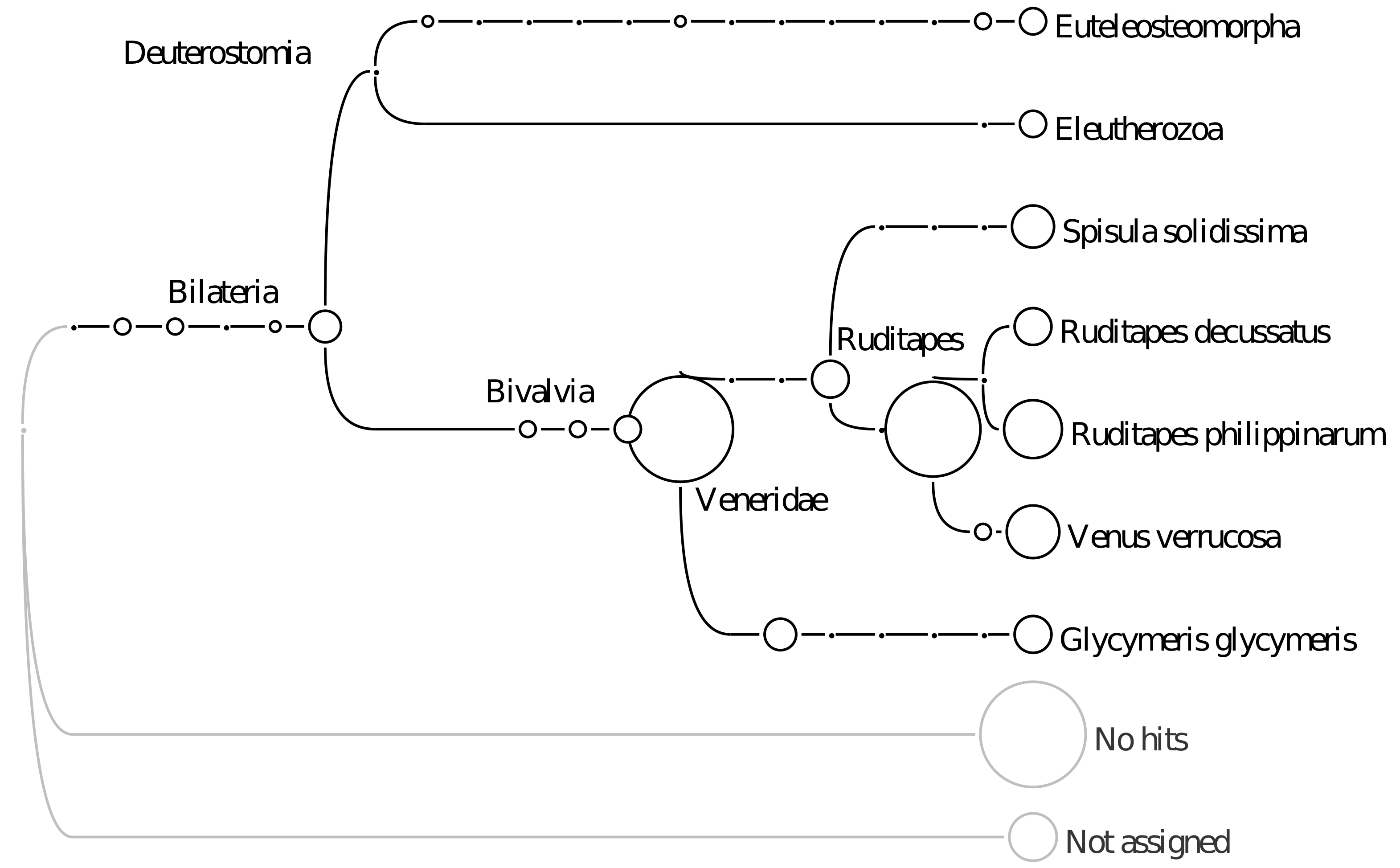

Supplement: Supplementary file 4 — Supplementary Information 4. [file 41598_2020_75984_MOESM4_ESM.tiff]
