# Supplementary material for: Omics approaches for conservation biology research on the bivalve Chamelea gallina
Source: Sci Rep. 2020 Nov 5;10:19177. doi: 10.1038/s41598-020-75984-9 (PMC7645701; doi:10.1038/s41598-020-75984-9)
Supplement: Supplementary file 5 — Supplementary Information 5. [file 41598_2020_75984_MOESM5_ESM.docx]

| **performed comparisons** | **DEGs** | **Number of DEGs** |
| --- | --- | --- |
| SM_autumn vs spring | upregulated | 755 |
|  | downregulated | 731 |
|  | total | 1,486 |
| S_ autumn vs spring | upregulated | 848 |
|  | downregulated | 903 |
|  | total | 1,751 |
| S vs SM_spring | upregulated | 320 |
|  | downregulated | 302 |
|  | total | 622 |
| S vs SM_autumn | upregulated | 635 |
|  | downregulated | 342 |
|  | total | 977 |

**Supplementary Table S1. Differentially Expressed Genes (DEGs).** Number of DEGs after filtering of p-adjusted <0.01 from DESeq2 counts.
