# Supplementary material for: Omics approaches for conservation biology research on the bivalve Chamelea gallina
Source: Sci Rep. 2020 Nov 5;10:19177. doi: 10.1038/s41598-020-75984-9 (PMC7645701; doi:10.1038/s41598-020-75984-9)
Supplement: Supplementary file 6 — Supplementary Information 6. [file 41598_2020_75984_MOESM6_ESM.docx]

**
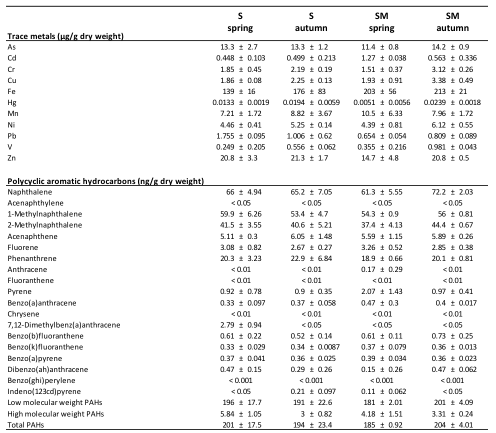
**

**Supplementary Table S2. Concentrations of chemicals measured in clam tissues**. Data are given as mean values ± standard deviations (n = 5).
