# Supplementary material for: Omics approaches for conservation biology research on the bivalve Chamelea gallina
Source: Sci Rep. 2020 Nov 5;10:19177. doi: 10.1038/s41598-020-75984-9 (PMC7645701; doi:10.1038/s41598-020-75984-9)
Supplement: Supplementary file 7 — Supplementary Information 7. [file 41598_2020_75984_MOESM7_ESM.docx]

| **SENIGALLIA (S)** | **Temperature** | **Salinity** | **Chlorophyll** |
| --- | --- | --- | --- |
| Jan | 10.10 ± 0.04 | 36.43 ± 0.05 | 1.25 ± 0.12 |
| Feb | 9.22 ± 0.09 | 35.58 ± 0.11 | 0.73 ± 0.07 |
| Mar | 9.33 ± 0.17 | 35.33 ± 0.14 | 1.05 ± 0.10 |
| **Winter** | 9.56 ± 0.08 | 35.79 ± 0.08 | 1.02 ± 0.06 |
| Apr | 13.90 ± 0.42 | 36.52 ± 0.02 | 0.96 ± 0.13 |
| May | 20.66 ± 0.30 | 34.94 ± 0.10 | 0.66 ± 0.06 |
| Jun | 24.75 ± 0.10 | 35.14 ± 0.08 | 0.51 ± 0.05 |
| **Spring** | 19.78 ± 0.50 | 35.53 ± 0.08 | 0.71 ± 0.05 |
| Jul | 26.99 ± 0.16 | 36.34 ± 0.02 | 0.34 ± 0.03 |
| Aug | 28.63 ± 0.17 | 36.52 ± 0.01 | 0.21 ± 0.03 |
| Sep | 25.10 ± 0.20 | 36.75 ± 0.03 | 0.28 ± 0.02 |
| **Summer** | 26.93 ± 0.18 | 36.53 ± 0.02 | 0.28 ± 0.02 |
| Oct | 19.95 ± 0.18 | 36.88 ± 0.02 | 0.45 ± 0.05 |
| Nov | 16.02 ± 0.29 | 37.25 ± 0.16 | 1.05 ± 0.10 |
| Dec | 11.82 ± 0.14 | 35.07 ± 0.01 | 1.05 ± 0.07 |
| **Autumn** | 15.93 ± 0.37 | 36.39 ± 0.11 | 0.85 ± 0.05 |

**Supplementary Table S3**. Mean values ± standard error of monthly temperature [°C], salinity [PSU] and chlorophyll-a [µg/l] at the S site in 2018.
