# Supplementary material for: Omics approaches for conservation biology research on the bivalve Chamelea gallina
Source: Sci Rep. 2020 Nov 5;10:19177. doi: 10.1038/s41598-020-75984-9 (PMC7645701; doi:10.1038/s41598-020-75984-9)
Supplement: Supplementary file 8 — Supplementary Information 8. [file 41598_2020_75984_MOESM8_ESM.docx]

| **SILVI MARINA (SM)** | **Temperature** | **Salinity** | **Chlorophyll** |
| --- | --- | --- | --- |
| Jan | 10.81 ± 0.04 | 37.34 ± 0.02 | 1.08 ± 0.06 |
| Feb | 9.96 ± 0.09 | 37.16 ± 0.07 | 0.80 ± 0.06 |
| Mar | 10.58 ± 0.13 | 36.31 ± 0.01 | 0.63 ± 0.06 |
| **Winter** | 10.47 ± 0.07 | 36.93 ± 0.05 | 0.84 ± 0.04 |
| Apr | 14.57 ± 0.38 | 36.44 ± 0.01 | 0.54 ± 0.05 |
| May | 20.95 ± 0.30 | 36.32 ± 0.04 | 0.26 ± 0.02 |
| Jun | 25.16 ± 0.10 | 35.68 ± 0.04 | 0.34 ± 0.03 |
| **Spring** | 20.23 ± 0.48 | 36.15 ± 0.04 | 0.38 ± 0.02 |
| Jul | 27.11 ± 0.15 | 36.82 ± 0.05 | 0.20 ± 0.02 |
| Aug | 28.51 ± 0.13 | 37.16 ± 0.02 | 0.15 ± 0.01 |
| Sep | 25.83 ± 0.22 | 37.36 ± 0.05 | 0.16 ± 0.01 |
| **Summer** | 27.17 ± 0.15 | 37.11 ± 0.03 | 0.17 ± 0.01 |
| Oct | 20.53 ± 0.22 | 37.94 ± 0.01 | 0.36 ± 0.03 |
| Nov | 16.69 ± 0.19 | 38.07 ± 0.02 | 0.65 ± 0.07 |
| Dec | 12.69 ± 0.19 | 37.44 ± 0.09 | 0.90 ± 0.07 |
| **Autumn** | 16.64 ± 0.36 | 37.81 ± 0.04 | 0.64 ± 0.04 |

**Supplementary Table S4**. Mean values ± standard error of seasonal temperature [°C], salinity [PSU] and chlorophyll-a [µg/l] at the SM site in 2018.
