# Supplementary material for: Omics approaches for conservation biology research on the bivalve Chamelea gallina
Source: Sci Rep. 2020 Nov 5;10:19177. doi: 10.1038/s41598-020-75984-9 (PMC7645701; doi:10.1038/s41598-020-75984-9)
Supplement: Supplementary file 9 — Supplementary Information 9. [file 41598_2020_75984_MOESM9_ESM.docx]

| **Temperature** | | **S** | | | | | | | |
| --- | --- | --- | --- | --- | --- | --- | --- | --- | --- |
|  |  | Winter | Spring | | Summer | | Autumn | | |
| **SM** | Winter | 0.488 |  | |  | |  | | |
|  | Spring |  | 0.974 | |  | |  | | |
|  | Summer |  |  | | 0.999 | |  | | |
|  | Autumn |  |  | |  | | 0.766 | | |
| **Salinity** | | **S** | | | | | | | |
|  |  | Winter | Spring | | Summer | | | Autumn | |
| **SM** | Winter | 0.000 |  | |  | | |  | |
|  | Spring |  | 0.000 | |  | | |  | |
|  | Summer |  |  | | 0.000 | | |  | |
|  | Autumn |  |  | |  | | | 0.000 | |
| **Chlorophyll** | | **S** | | | | | | | |
|  |  | Winter | | Spring | | Summer | | | Autumn |
| **SM** | Winter | 0.038 | |  | |  | | |  |
|  | Spring |  | | 0.000 | |  | | |  |
|  | Summer |  | |  | | 0.590 | | |  |
|  | Autumn |  | |  | |  | | | 0.006 |

**Supplementary Table S5**. P-values obtained from the ANOVA and Tukey test for the comparisons of the seasonal temperature [°C], salinity [PSU] and chlorophyll concentration [mg/l] between the study areas. p-values below the level of significance (p < 0.05) are highlighted in red.
