# Supplementary material for: Omics approaches for conservation biology research on the bivalve Chamelea gallina
Source: Sci Rep. 2020 Nov 5;10:19177. doi: 10.1038/s41598-020-75984-9 (PMC7645701; doi:10.1038/s41598-020-75984-9)
Supplement: Supplementary file 10 — Supplementary Information 10. [file 41598_2020_75984_MOESM10_ESM.docx]

|  | **S** | | | |  | **SM** | | | |
| --- | --- | --- | --- | --- | --- | --- | --- | --- | --- |
| **Temperature** | W | Sp | Su | A | **Temperature** | W | Sp | Su | A |
| W |  |  |  |  | W |  |  |  |  |
| Sp | ⚫ |  |  |  | Sp | ⚫ |  |  |  |
| Su | ⚫ | ⚫ |  |  | Su | ⚫ | ⚫ |  |  |
| A | ⚫ | ⚫ | ⚫ |  | Au | ⚫ | ⚫ | ⚫ |  |
| **Salinity** | W | Sp | Su | A | **Salinity** | W | Sp | Su | A |
| W |  |  |  |  | W |  |  |  |  |
| Sp | ⭘ |  |  |  | Sp | ⚫ |  |  |  |
| Su | ⚫ | ⚫ |  |  | Su | ⭘ | ⚫ |  |  |
| A | ⚫ | ⚫ | ⭘ |  | A | ⚫ | ⚫ | ⚫ |  |
| **Chlorophyll** | W | Sp | Su | A | **Chlorophyll** | W | Sp | Su | A |
| W |  |  |  |  | W |  |  |  |  |
| Sp | ⚫ |  |  |  | Sp | ⚫ |  |  |  |
| Su | ⚫ | ⚫ |  |  | Su | ⚫ | ⚫ |  |  |
| A | ⭘ | ⭘ | ⚫ |  | A | ⚫ | ⚫ | ⚫ |  |

**Supplementary Table S6.** Results from Tukey’s pairwise comparison among seasons (W: winter, Sp: spring, Su: summer, A: autumn) at the sampling stations of S (left columns) and SM (right columns). Full circles denote significant differences (p < 0.05), empty circles non-significant differences (p > 0.05). Only half of the symmetric panels is filled.
