# Supplementary material for: Omics approaches for conservation biology research on the bivalve Chamelea gallina
Source: Sci Rep. 2020 Nov 5;10:19177. doi: 10.1038/s41598-020-75984-9 (PMC7645701; doi:10.1038/s41598-020-75984-9)
Supplement: Supplementary file 11 — Supplementary Information 11. [file 41598_2020_75984_MOESM11_ESM.docx]

**Supplementary File S1. Detailed protocol for ecotoxicological and bioaccumulation analyses.**

**Ecotoxicological and chemical analyses**

For Condition Index (CI) calculation, 30 bivalves were cleaned and dissected for each sampling group. Whole tissues and shell were placed into a drying oven at 60 °C for 12 h and their dry weights determined through an analytical balance. The CI was calculated according to the formula: CI = [dry tissue weight (g)/dry shell weight (g)] × 100^85^.

Lysosomal membrane stability was evaluated in clam haemocytes as Neutral Red Retention Time (NRRT) using the cationic probe Neutral Red (NR). 5 specimens were individually analysed for each sampling site and period. Haemocytes were incubated on a glass slide with a NR working solution (2µl/ml filtered sea water from a stock solution of 20 mg NR dye dissolved in 1 ml of dimethyl sulfoxide) and microscopically examined at 20 min intervals to determine the time at which 50% of cells had lost into the cytosol the dye previously taken up by lysosomes.

AChE activity was spectrophotometrically assayed in clams hemolymph using the Ellman’s reaction, with acetylthiocholine and 5,5-dithiobis-2-nitrobenzoic acid (DTNB).

The study of antioxidant responses was performed on clams digestive glands through the analyses of single antioxidants (enzymatic activities of catalase, glutathione S-transferases, glutathione reductase, glutathione peroxidases and the levels of total glutathione) and the measurement of the total antioxidant capacity (TOSC assay towards peroxyl and hydroxyl radicals).

For enzymatic antioxidants, samples of digestive glands were homogenized (1:5 w:v ratio) in 100 mM K-phosphate buffer (pH 7.5), with NaCl 2.5% and 0.1 mM phenylmethylsulphonyl fluoride (PMSF), 0.008 TIU/mL aprotinin, 1 µg/mL leupeptin, 0.5 µg/mL pepstatin as protease inhibitors. After centrifugation at 110000 ×g for 1 h at 4 °C, the supernatant (cytosolic fraction) was recovered and stored at -80 °C for enzymatic assays. The measurements were made with a Varian (model Cary 3) spectrophotometer at a constant temperature of 18 °C. Catalase (CAT) was measured by the decrease in absorbance at 240 nm (extinction coefficient, ε = 0.04 mM^-1^ cm^-1^) due to the consumption of hydrogen peroxide, H_2_O_2_ (12 mM H_2_O_2_ in 100 mM K-phosphate buffer pH 7.0). Glutathione S-transferases (GST) were determined at 340 nm using 1-chloro-2,4-dinitrobenzene (CDNB) as substrate. The assay was carried out in 100 mM K-phosphate buffer pH 6.5, 1.5 mM CDNB, 1 mM GSH (ε = 9.6 mM^-1^ cm^-1^). Glutathione reductase (GR) was determined from NADPH oxidation during the reduction of oxidized glutathione, GSSG (λ = 340 nm, ε = 6.22 mM^-1^ cm^-1^). The final assay condition was 100 mM K-phosphate buffer pH 7.0, 1 mM GSSG, and 60 mM NADPH. Glutathione peroxidases (GPx) activities were assayed in a coupled enzyme system where the GSSG, produced by GPx, is reduced to GSH by glutathione reductase with consumption of NADPH, which is monitored as decrease of absorbance at 340 nm (ε = 6.22 mM^-1^ cm^-1^). The reaction is performed in 100 mM K-phosphate buffer pH 7.5, 1 mM EDTA, 1mM dithiothreitol, 2 mM GSH, 1 unit glutathione reductase, 0.24 mM NADPH, and 0.5 mM H_2_O_2_ (as substrate for Se-dependent GPx isoforms) or 0.8 mM cumene hydroperoxide (as substrate for the sum of Se-dependent and Se-independent forms).

Levels of total glutathione (tGSH) in the digestive glands were measured after homogenization (1:5, w/v ratio) in 5% sulphosalicilic acid with 4mM EDTA. Samples were maintained for 45 min on ice for deproteinization and centrifuged at 37000×g for 15 min. The resulting supernatants were enzymatically assayed as previously reported in Akerboom and Sies (1981). Calibration was performed using reduced glutathione (GSH) standards.

The total oxyradical scavenging capacity (TOSC) assay measure the overall capability of cellular antioxidants to absorb different forms of artificially generated oxyradicals, thus inhibiting the oxidation of 0.2 mM α-keto-γ-methiolbutyric acid (KMBA) to ethylene gas (Regoli and Winston, 1998). Peroxyl radicals (ROO•) were generated by the thermal homolysis of 20 mM 2,2’-azo-bis-(2-methylpropionamidine)-dihydrochloride (ABAP) in 100 mM K-phosphate buffer, pH 7.4. Hydroxyl radicals (•OH) were produced by the Fenton reaction of iron-EDTA (1.8 mM Fe^3+^, 3.6 mM EDTA) plus ascorbate (180 mM) in 100 mM K-phosphate buffer. Under these conditions, the different oxyradicals produced quantitatively similar yields of ethylene in control reactions, thus allowing the comparison of the relative efficiency of cellular antioxidants toward a quantitatively similar radical flux. Ethylene formation in control and sample reactions was analysed at 12 min time intervals (total time: 96 min) by gas-chromatographic analyses and the TOSC values are quantified from the equation: TOSC = 100 - (ʃSA/ʃCA × 100), where ʃSA and ʃCA are the integrated areas calculated under the kinetic curves for samples (SA) and control (CA) reactions. For all the samples, a specific TOSC (normalized to content of protein) was calculated by dividing the experimental TOSC values by the relative protein concentration contained in the assay.

Metallothioneins (MTs) were analyzed in clams digestive glands after acidic ethanol/chloroform fractionation of tissues homogenates, and spectrophotometric quantification using reduced glutathione (GSH) as standard^98^.

Protein concentrations were measured according to Lowry method, using bovine serum albumin (BSA) as standard.

Trace metals in clams were measured after digestion under pressure with nitric acid and hydrogen peroxide (7:1) with microwave^99^. Arsenic, cadmium, chromium, copper, iron, manganese, nickel, lead, vanadium, and zinc were analyzed by atomic absorption spectrophotometry, with flame (Varian, Spectraa 220FS) and flameless atomization (Varian Spectraa 240Z); the mercury content was quantified by cold vapour atomic absorption spectrometry (Varian, VGA-76, Vapour Generator Accessory and Cetac QuickTrace M-6100 Mercury Analyzer). Concentrations of polycyclic aromatic hydrocarbons (PAHs) were determined in mussels after methanolic extraction with microwave, solid-phase purification and HPLC analyses with fluorimetric detection^96^. The PAHs were identified according to the retention times of an appropriate pure standards solution (EPA 610 Polynuclear Aromatic Hydrocarbons Mix), and classified as low molecular weight (LMW: naphthalene, acenaphthylene, 1-metylnaphthalene, 2-metylnaphthalene, acenaphthene, fluorene, phenanthrene, anthracene) or high molecular weight (HMW: fluoranthene, pyrene, benzo[a]anthracene, chrysene, 7,12-dimetylbenzo[a]anthracene, benzo[b]fluoranthene, benzo[k]fluoranthene, benzo[a]pyrene, dibenzo[a,h]anthracene, benzo[g,h,i]perylene, indeno[1,2,3,c,d]pyrene).

In all chemical analyses, quality assurance and control were monitored by processing blank and reference standard materials (mussel tissue Standard Reference Material [SRM] 2977, National Institute of Standards and Technology). The concentrations obtained from these SRM analyses were always within the 95% confidence intervals of the certified values. The water content in tissues was determined in all of the samples, and the concentrations were expressed as μg/g dry weight (dw) for trace metals, and ng/g dw for PAHs (mean values ± standard deviations, n=5).
