# Supplementary material for: Omics approaches for conservation biology research on the bivalve Chamelea gallina
Source: Sci Rep. 2020 Nov 5;10:19177. doi: 10.1038/s41598-020-75984-9 (PMC7645701; doi:10.1038/s41598-020-75984-9)
Supplement: Supplementary file 13 — Supplementary Information 13. [file 41598_2020_75984_MOESM13_ESM.pdf]

Supporting Information

Omics approaches for conservation biology research on the bivalve *Chamelea gallina*

Federica Carducci†, Maria Assunta Biscotti†, Emiliano Trucchi, Maria Elisa Giuliani, Stefania Gorbi, Alessandro Coluccelli, Marco Barucca†, Adriana Canapa†*

Supplementary Fig. S1. Performed comparisons in gene expression analyses. Schematic representation of pairwise comparisons performed using DESeq2 to obtain differential expressed genes.

Supplementary Fig. S2. PCA plots. Principal Component Analyses of gene expression profiles obtained for each pairwise comparison made between sampling times (A and B) and sampling sites (C and D).

Supplementary Fig. S3. **Robustness of *fineRADstructure* inference**. Robustness of *fineRADstructure* inference to intra-locus variation and missing data per individual. Distribution of the number of alleles (A) and SNPs (B) per locus across all loci in the dataset; the threshold of 45 SNPs in a locus (red vertical line) is shown. Proportion of missing data per individual (C); the threshold of 25% of missingness per individual is shown (red horizontal line); note the allele drop-out in individual from the Tyrrhenian population (N). Co-ancestry matrixes inferred by *fineRADstructure* using all individuals but only loci with less than 45 SNPs (E), all individuals and all loci (F), only individuals with less than 25% missing data and only loci with less than 45 SNPs (G), only individuals with less than 25% missing data and all loci (H); panel g corresponds to Figure 2 in the main text. Number of loci for which any two individuals are the closest relatives is coded according to the palette in the middle (scale bars are towards the relative matrix). The Adriatic population includes samples from Ma, Mo, S and SM, whereas only N is the locality from the Tyrrhenian sea. Few more-related-than-average individuals are highlighted.

Supplementary Fig. S4. Taxonomy identification using MEGAN. Taxonomy identification of the 300 loci showing a significant match in the GenBank *nt* database (*epsilon* < 1E-04) as summarized in MEGAN. Circle size for each taxon is proportional to the number of matching loci (excluding No hits and Not Assigned).

Supplementary Table S1. Differentially Expressed Genes (DEGs). Number of DEGs after filtering of p-adjusted <0.01 from DESeq2 counts.

Supplementary Table S2. Concentrations of chemicals measured in clam tissues. Data are given as mean values ± standard deviations (n = 5).

Supplementary Table S3. Mean values ± standard error of monthly temperature [°C], salinity [PSU] and chlorophyll-a [µg/l] at the S site in 2018.

Supplementary Table S4. Mean values ± standard error of seasonal temperature [°C], salinity [PSU] and chlorophyll-a [µg/l] at the SM site in 2018.

Supplementary Table S5. P-values obtained from the ANOVA and Tukey test for the comparisons of the seasonal temperature [°C], salinity [PSU] and chlorophyll concentration [mg/l] between the study areas. p-values below the level of significance ($p < 0.05$) are highlighted in red.

Supplementary Table S6. Results from Tukey's pairwise comparison among seasons (W: winter, Sp: spring, Su: summer, A: autumn) at the sampling stations of S (left columns) and SM (right columns). Full circles denote significant differences ($p < 0.05$), empty circles non-significant differences ($p > 0.05$). Only half of the symmetric panels is filled.

Supplementary File S1. Detailed protocol for ecotoxicological and bioaccumulation analyses.

Supplementary File S2. Custom python scripts *vcf tools*.
